# Modeling cancer with bacteria-integrated tumor microenvironments using biomaterials: Emerging concepts and opportunities

**DOI:** 10.1016/j.mtbio.2026.103330

**Published:** 2026-06-09

**Authors:** Keuna Jeon, Uijin Kim, Chang-Hun Ji, Meenakshi Kamaraj, Noah Zachary Laird, Zhikun Wang, Hongxiao Yu, Menekse Ermis, Ruby May A. Sullan, Xiling Shen, Natashya Falcone

**Affiliations:** a Terasaki Institute for Biomedical Innovation, Los Angeles, CA, 91367, USA; b Leibniz Institute for Natural Product Research and Infection Biology, Hans Knöll Institute (HKI), Beutenbergstraße 11a, Jena, 07745, Germany; c Department of Bioengineering, Indian Institute of Science, Bangalore, Karnataka, 560012, India; d Department of Pediatrics, David Geffen School of Medicine, University of California, Los Angeles, Los Angeles, USA; e Department of Physical and Environmental Sciences, University of Toronto Scarborough, 1265 Military Trail, Toronto, Ontario, Canada; f Department of Chemistry, University of Toronto, 80 St. George St., Toronto, Ontario, Canada

**Keywords:** Tumor microbiome, Tumor microenvironmental, Biomaterials, Cancer modeling cancer-bacteria interactions

## Abstract

The tumor microenvironment (TME) is a dynamic and heterogeneous ecosystem in which cancer, stromal, immune, and physicochemical components collectively regulate disease progression and therapeutic response. Recent evidence further indicates that intratumoral bacteria are active contributors to tumor metabolism, immune modulation, and treatment outcomes, revealing a previously underexplored multi-kingdom dimension of solid tumors. However, mechanistic understanding of tumor–microbe interactions remain limited by the absence of experimental platforms that integrate microbial components into physiologically relevant and controllable tumor models. Here, we propose bacteria-integrated tumor microenvironments as an emerging bioengineering framework for modeling cancer as a multi-kingdom system with biomaterials. We first lay out the existence of bacteria in our body and outline key design principles for these systems, including control of microbial localization, nutrient and oxygen gradients, and interkingdom signaling within engineered matrices. We further discuss applications in studying microbial contributions to therapeutic resistance, evaluating engineered bacterial therapies, and developing patient-specific tumor–microbiome models for precision oncology. Finally, we highlight challenges in standardizing multi-kingdom tumor platforms and integrating them with advanced imaging, sequencing, and computational tools. Collectively, bacteria-integrated TME establish a new paradigm for engineering cancer as a multi-kingdom system with translational potential in oncology.

## Graphical abstract

**Figure:**
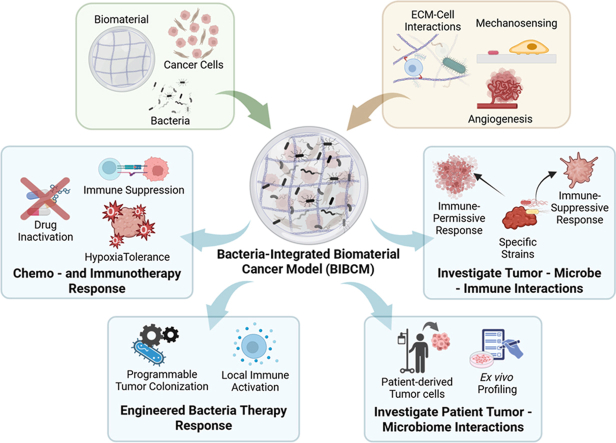

## 1 Introduction

The presence of bacteria within tumor microenvironments (TME) have been recognized for over a century; however, the identification of specific intracellular bacterial species associated with distinct tumor types has only recently become possible due to advances in sequencing technologies and improved detection of low biomass microbial populations [[1], [2], [3]]. The frequent failures of new cancer therapeutics in clinical trials suggest that critical components of the TME remain insufficiently modeled in preclinical models [4,5]. The TME is a highly complex and dynamic system composed of physical, chemical, and biological factors, including extracellular matrix (ECM) stiffness, soluble signaling factors such as cytokines and growth factors, and diverse cell populations. Importantly, the TME comprises a multi-kingdom ecosystem, encompassing interacting biological entities from distinct kingdoms including bacteria, fungi, viruses, and host (mammalian) cells that collectively influence tumor behavior and therapeutic response. Increasing evidence indicates that tumor-associated bacteria represent an underappreciated layer influencing cancer progression and therapeutic response [6]. In particular, intratumoral bacteria were shown to mediate the resistance to chemotherapeutic drugs [4] and modulate responses to immune checkpoint blockades [5]. Microbial components have emerged as additional modulators capable of reshaping tumor metabolism, cancer progression, immune responses, and therapeutic responses [7]. Furthermore, variations in microbial composition and diversity within the tumors have been linked to differences in tumor progression, metastatic potential of tumor, and patient outcomes [8]. Collectively, these findings highlight the importance of microbial factors into models of the TME to better understand cancer biology and improve therapeutic development in cancer treatments.

While two- and three-dimensional co-culture systems have been developed to study interactions between mammalian cells and bacteria for oral and gastrointestinal microenvironment engineering [9], these approaches remain limited in their ability to recapitulate the structural and functional complexity of solid tumors. A major challenge in modeling tumor–bacteria interactions lie in the differences in growth kinetics between microbial and mammalian cells, often resulting in uncontrolled bacterial overgrowth that disrupts stable co-culture systems. To address these challenges, microfluidics systems have been employed where the excess growth bacterial species can be washed away with the flow [10]. However, despite these advances, microfluidics systems alone often lack the capacity to fully reproduce the natural three-dimension (3D) architecture, mechanical heterogeneity, and biochemical gradients that characterize native tumor tissues. Hydrogel-based biomaterials offer a promising strategy for constructing next-generation cancer models that incorporate both TME and microbiome components [11,12]. Recent perspectives have emphasized the need for systematic integration of biomaterial engineering and tumor microbiome modeling to enable more clinically relevant investigation of host–microbe interactions within the TME [3]. These materials provide tunable physical and biochemical properties that can mimic key features of the TME while enabling spatially controlled integration of specific microbial species [13,14]. These platforms support the investigation of tumor–microbe interactions under physiologically relevant conditions and facilitate *in vitro* drug screening applications with improved throughput [15]. Incorporating microbial components into biomaterial-based organoid models represents a critical step towards more accurately recapitulating *in vivo* TME and bridging the gap between preclinical studies and clinical outcomes.

In this review, we focus specifically on how biomaterial-based platforms can enable mechanistic investigation of tumor–microbe interactions. We first distinguish the systemic roles of the gut microbiome from the localized effects of intratumoral bacteria, then evaluate existing experimental models and their limitations. Finally, we highlight emerging biomaterial design principles and technologies that allow controlled integration of microbial components into TME, enabling new opportunities to study therapeutic resistance, engineer bacteria-based therapies, and develop patient-specific tumor–microbiome platforms. This review specifically focuses on biomaterial-enabled bacteria-integrated TME models as an emerging engineering framework for studying cancer as a multi-kingdom system. Rather than broadly reviewing microbiome associations in cancer or general TME engineering, we emphasize how biomaterials can be used to spatially and mechanically control host–microbe interactions within physiologically relevant tumor models. We discuss current limitations in existing tumor microbiome models, key biomaterial design principles for integrating microbial populations into tumor systems, and emerging applications in mechanistic cancer biology, microbial therapeutics, and precision oncology.

## 2 Biological foundations of the microbiome: gut vs. intratumoral microbiota

**Fig. 1:**
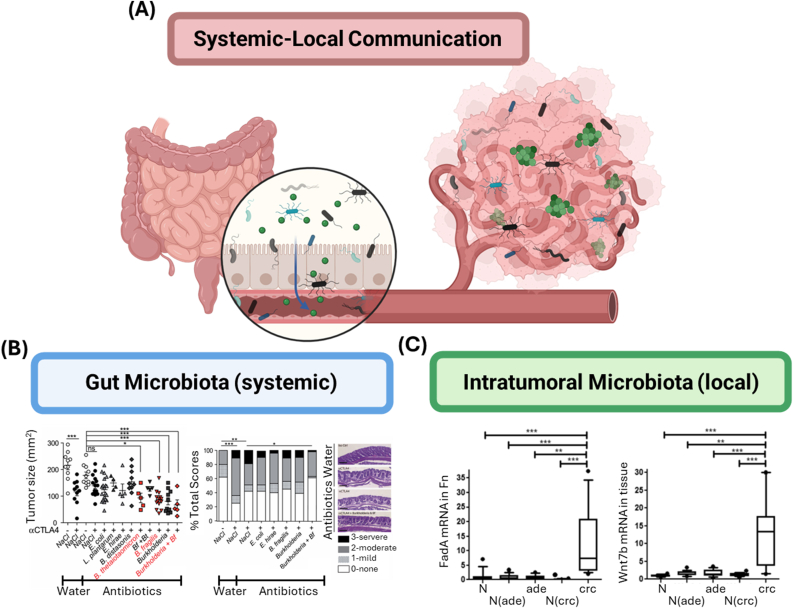
Distinct roles of gut and intratumoral microbiota in cancer-associated processes. **(A)** Schematic illustration of proposed communication pathways between gut microbiota, systemic circulation, immune signaling, and the tumor microenvironment. Potential routes of interaction include microbial metabolites, immune modulation, and bacterial translocation. **(B)** Representative outcomes associated with CTLA-4 blockade therapy in mice with different gut microbiota compositions, including tumor size measurements (left), histopathological scoring of colonic mucosa(middle), and representative colon histology images (right). (Fig. 3a, reproduced with permission from Vétizou et al., *Science*, 2016, © American Association for the Advancement of Science(AAAS)) [17]. **(C)** Expression analysis of β-catenin and Wnt signaling-associated genes in healthy tissue, precancerous adenomas, and carcinoma samples. Relative expression levels of *FadA* (left) and *Wnt7b* (right) are shown across tissue groups. (Fig. 7.b,c reproduced from Rubinstein et al., 2014, *PLOS ONE*, licensed under CC BY 4.0) [18].

The microbiome contributes to tumor biology through both systemic and local mechanisms [16]. While the gut microbiota has been extensively studied as a regulator of systemic immunity and therapeutic response, bacteria residing within tumors represent a distinct and spatially localized component of the TME. These two microbial compartments exert nonredundant but interconnected effects on cancer progression. In this section, we distinguish the roles of gut and intratumoral microbiota to clarify their respective contributions to tumor biology (Fig. 1) [17,18].

### 2.1 The gut microbiome

The gut microbiota is a diverse and dynamic microbial ecosystem composed of bacteria, fungi, viruses, and archaea that plays a central role in immune homeostasis, metabolism, and epithelial barrier function. Through continuous interactions with host immune and metabolic systems, the gut microbiome establishes systemic signaling networks that extend beyond the gastrointestinal tract and influence distant tissues, including tumors [[19], [20], [21]]. Gut microbiota can regulate anti-tumor immunity by modulating immune cell recruitment, activation, and metabolic signaling through circulating microbial products. Certain microbial compositions promote dendritic cell activation and Th1/cytotoxic T-cell responses that enhance immune checkpoint blockade therapies, including CTLA-4 inhibitors. In contrast, gut microbiota disruption by antibiotics, chemotherapy, aging, or disease impairs antitumor immunity and therapeutic efficacy (Fig. 3A) [19,22,23]. In addition, gut microbiota-derived metabolites exert context-dependent effects, where some support immune homeostasis while others enhance tumor survival and DNA repair, thereby reducing treatment efficacy [22,24,25]. Collectively, these findings position the gut microbiota as a systemic immunometabolic regulator capable of either enhancing or suppressing anti-tumor immunity depending on microbial composition and host conditions [19,22,26]. However, unlike the gut microbiome, intratumoral microbes function locally within the tumor microenvironment, where they directly interact with cancer, stromal, and immune cells in spatially confined niches. As a result, understanding intratumoral microbial behavior requires experimental systems capable of modeling localized host–microbe interactions within physiologically relevant tumor architecture.

### 2.2 The tumor microbiome

On the other hand, systemic modulation differs fundamentally from local microenvironmental regulation within tumors. Intratumoral bacteria interact directly with tumor and immune cells within the TME [1]. They function locally within the TME, where bacteria and fungi residing intracellularly in cancer and immune cells directly influence the tumor behavior [1,27,28]. These microbes can alter tumor cell transcriptional programs, increase signaling plasticity, and reshape immune cell states in a spatially restricted manner [[29], [30], [31]]. Intratumoral microbial populations have been shown to promote genomic instability through induction of oxidative stress and DNA double-strand breaks while activating oncogenic pathways such as β-catenin and Wnt signaling to enhance tumor cell proliferation, invasion, and metastasis [18,31]. Furthermore, tumor-resident microbes frequently contribute to immune evasion by suppressing cytotoxic CD8^+^ T cells and natural killing (NK) cell activity and promoting immunosuppressive macrophage phenotypes [27]. Unlike gut microbiota, which modulate therapy through systemic immune modulation, intratumoral microbes can directly reduce therapeutic efficacy [27,32]. For example, they can play a role in enzymatically inactivating chemotherapeutic agents, such as Gammaproteobacteria-mediated gemcitabine, highlighting their role as localized drivers of oncogenic signaling, immune evasion, genomic instability, and drug resistance [4,23,33].

Although studies have established that bacteria integrated into tumor ecosystems, defining their mechanistic contributions remains challenging. Current insights come from correlative sequencing analyses or *in vivo* observations, both of which offer limited control over microbial localization, abundance, and interactions with host cells. Therefore, experimental model systems are essential for dissecting tumor–microbe interactions under well-defined conditions.

## 3 Experimental models of the tumor microbiome

**Fig. 2:**
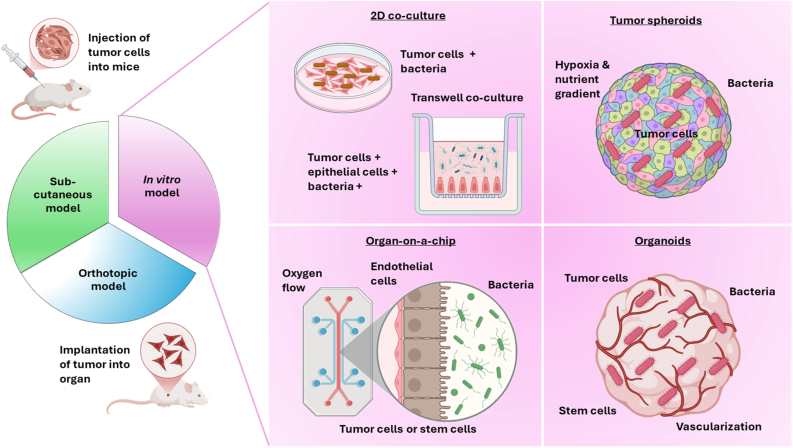
Experimental platforms used to investigate tumor-microbiome interactions. Overview of commonly used *in vivo* and *in vitro* tumor microbiome models. *In vivo* systems include subcutaneous and orthotopic tumor models. *In vitro* platforms include 2D co-culture systems, tumor spheroids, organ-on-a-chip systems, and organoids. These platforms provide distinct experimental environments for studying microbial localization, tumor-associated responses, and microenvironmental interactions.

Experimental models are essential for dissecting tumor–microbe interactions under controlled conditions. Existing systems span *in vivo* models and a range of *in vitro* platforms, each offering distinct advantages and limitations in capturing tumor complexity and microbial dynamics. Currently, *in vivo* models are the most used models, however, *in vitro* models provide higher resolution and other advantages. These systems range from simplified two-dimensional (2D) co-culture platforms to complex 3D and microfluidic models and have been developed and employed to study these interactions to predict tumor cell survival and progression or suppression (Fig. 2) [35,36]. In this section, we evaluate these models with respect to their ability to represent spatial organization, microenvironmental control, and host–microbe interactions.

**Fig. 3:**
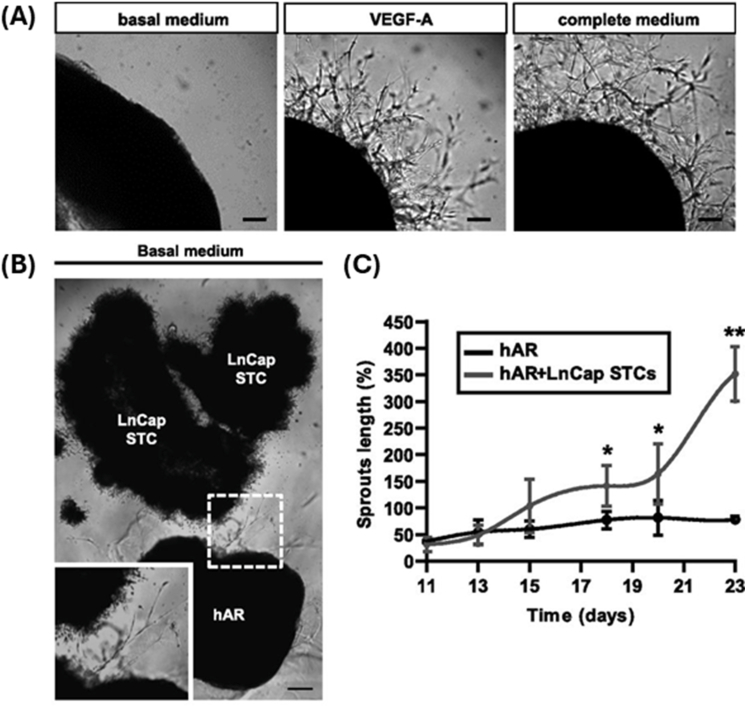
Angiogenic responses in human arterial ring (hAR) coculture systems. **(A)** Representative images of human arterial rings (hAR) for 15 days treated with basal medium (i.e. lacking growth factors), basal medium with VEGF-A, or complete medium (containing a variety of growth factors) with scale bar of 200 μm. **(B)** Representative images of hAR cocultured with tumor spheroids for 30 days in basal medium with scale bar of 400 μm. and **(C)** Quantification of angiogenic outgrowth length over time in hARs cultures with or without tumor spheroids [34]. (Figs. 2a and 6a,c, reproduced with permission from Seano et al., 2013).

### 3.1 Two-dimensional (2D) models

Conventional 2D cell culture systems have long been used to study cancer progression and therapeutic responses due to their simplicity and reproducibility. However, these models rely heavily on immortalized cell lines that fail to capture tumor heterogeneity, tissue architecture, and the hypoxic conditions characteristic of solid tumors. Additionally, long-term use of culture conditions can alter cellular phenotypes, limiting physiological relevance. Despite these limitations, 2D co-culture systems have provided mechanistic insights into cancer–microbiome interactions. Previous studies demonstrated the feasibility of co-culturing human cell lines with bacteria to investigate metabolic, proliferative, and invasive effects. For example, Rubinstein et al., demonstrated that *Fusobacterium nucleatum* binds to E-cadherin on epithelial cells through FadA adhesin, promoting bacterial invasion and stimulating oncogenic signaling pathways in colorectal cancer (CRC) cells [18]. Other studies examining intracellular interactions revealed that *Escherichia coli* (*E. coli*) can replicate within the cytoplasm of HeLa cells, with bacterial growth influenced by host cell nutrient levels (amino acid content), pH, and osmotic conditions, leading to mutualistic growth of bacteria and host cells [37]. Transwell systems have also been widely employed to physically separate epithelial cells from immune cells or bacteria while allowing the exchange of soluble factors. However, these conventional methods do not recapitulate the 3D structure, mechanical properties, or spatial gradients of native tumor tissues, limiting their ability to model complex tumor-microbiome interactions.

### 3.2 Tumor spheroids

Tumor spheroids represent 3D *in vitro* model formed by self-assembled cancer cell aggregates that enable enhanced cell–cell and cell–matrix interactions. Compared to 2D cultures, spheroids more closely mimic native tumor architecture, including the presence of oxygen and nutrient gradients, hypoxic cores, and dense ECM deposition, which acts as a protective barrier against external stimuli and contributes to the maintenance of cell viability [38,39]. These features allow spheroids to better reproduce physiological gene expression patterns and cellular function [40]. Importantly, spheroids provide a microenvironment that can support microbial viability. For example, CRC spheroids generated from HT-29 and HCT116 cell lines have been shown to sustain the proliferation of CRC-associated *F. nucleatum* and maintain bacterial viability in co-culture for at least 48 h [41]. This capability is largely attributed to the intrinsic oxygen gradient within spheroids featuring an oxygen-rich periphery and a hypoxic core which creates a favorable microenvironment for anaerobic bacteria. By incorporating *F. nucleatum* into these spheroid models, the study provided important insights into bacteria-mediated modulation of the TME and its potential contribution to cancer progression [41]. Advanced biofabrication strategies have further enhanced spheroid modeling capabilities. Han et al. introduced 3D printing components to establish a relevant vascularized tumor model [42]. A bioprinted tissue layer composed of fibroblasts and enterocytes was cultured to create a vascularized microenvironment supporting glioblastoma spheroids, which exhibited accelerated growth and early vascular infiltration. Furthermore, Kasper et al. showed that 3D tumor spheroids support anaerobic bacteria while enabling transcriptomic and metabolomic analyses, and observed biofilm-like bacterial structures within the spheroid microenvironment [41]. Collectively, 3D spheroid models bridge the gap between conventional 2D *in vitro* systems and clinically relevant tumor behavior by enabling more physiologically predictive studies of tumor architecture, microbial interactions, and therapeutic response. Despite these advances, challenges remain including limited spheroid lifespan, variability in nutrient and oxygen gradients, and technical difficulties in extracting and characterizing microbial populations within densely packed cellular structures.

### 3.3 Organoids

Organoids are stem-cell or patient-derived 3D culture systems that recapitulate key aspects of tissue organization, cellular diversity, and physiological function compared to immortalized cell lines or spheroids [43,44]. They can be used for understanding cancer development, drug testing, and host-microbiome interactions *in vitro* [45]. Generated from adult stem cells (ASCs), induced pluripotent stem cells (iPSCs), or embryonic stem cells (ESCs), organoids are typically embedded within a protein-rich ECM hydrogel such as Matrigel or basement membrane extract. These systems enable long-term culture and maintain tissue-specific architecture, making them powerful tools for disease modeling and precision medicine as they enable sustained experimental studies [46]. Microbial integration into organoid systems has provided valuable insights into host–microbe interactions in cancer and infection contexts. For example, Bartfeld et al. reported the development of human gastric organoid models with *Helicobacter pylori* to study infection and pathology where organoids retained tissue-specific characteristics [47]. Upon infection, *H. pylori* established close contact with the epithelial cells, remained viable, and induced localized infection, leading to activation of NF-κB signaling pathway, a central regulator of inflammatory and innate immune responses that controls cytokine production, epithelial survival, and barrier function. Cells from both pit and gland lineages exhibited responsive behavior. Similarly, studies using human intestinal organoids exposed to *Clostridium difficile* toxins revealed microbial modulation of epithelial integrity and microbiome composition, leading to disease conditions [48,49]. Additionally, Pleguezuelos-Manzano et al. demonstrated that exposure of human intestinal organoids to genotoxic pks ^+^ *E*. *coli* induces a distinct mutational signature associated with colorectal cancer, providing direct evidence that patient-relevant microbial strains can drive tumorigenic genomic alterations [50]. Apart from disease condition systems, patient-derived tumor organoids combined with patient-specific microbial populations represent an emerging platform with significant translational potential.

### 3.4 Organ-on-a-chip (OoC)

Organ-on-a-Chip (OoC) platforms are microengineered systems designed to replicate key physiological features of native tissues, including mechanical forces, vascular flow, biochemical cues, and gradients of pH, nutrients, and oxygen [51,52]. These systems enable precise control over environmental parameters such as oxygen tension, nutrient supply, and shear stress, making them particularly suitable for studying dynamic host–microbe interactions. Several OoC models have been developed to support co-culture of human cells including spheroids or organoids, ECM or tissue-mimicking hydrogel, with anaerobic bacteria cultures and enable the oxygen and chemokine gradients [53]. OoC platforms typically will have two or more compartmentalization achieved through membranes or hydrodynamic features (micropillars, narrow gaps) and embed cell-laden hydrogel, perfusable media and air flow along with either direct or indirect culture of bacteria [54,55]. The hydrogels play a significant role in influencing the cell function and maintaining their phenotype, commonly used are collagen type 1, fibrin, laminin, Matrigel, polyethylene glycol and polyacrylates [56,57]. These hydrogels serve as structural component as well as provide biochemical cues, formation gradients [58,59]. The recently established HumiX platform enables controlled communication between intestinal epithelial cells and commensal bacteria such as *Bacteroides caccae* under physiologically relevant gastrointestinal conditions [60]. Similarly, mucosal anoxic–oxic interface chips recreate oxygen gradients across the colonic epithelium *in vivo*, supporting the survival of obligate anaerobes while maintain epithelial viability and function [61]. In microfluidic culture systems, continuous laminar flow of media through channels lined with epithelial cells establishes controlled hydrodynamic conditions. The volumetric flow rate is critical, as it governs shear stress magnitude a key parameter influencing cell adhesion, phenotype maintenance, and resistance to detachment [62]. Beyond flow dynamics, several design parameters collectively influence cell retention and function including the polymeric network material and its surface properties, device geometry and channel architecture, tissue-mimetic topography of internal channel surfaces, and in polymicrobial systems, intercellular interactions and biofilm formation. Together, these factors create a biomimetic microenvironment that sustains long-term culture viability and prevents cell washout [63]. More advanced gut microbiome-on-a-chip systems allow stable co-culture of microbial communities such as *Bacteroides fragilis* alongside epithelial tissues. These technologies demonstrate the growing ability to model physiologically relevant tumor–microbiome interactions under controlled conditions and enable long-term culture of both human and microbial cells [64]. This platform uniquely supported the co-culture of metabolically opposing bacterial species (aerobic and anaerobic) in a single device while preserving epithelial barrier integrity a critical requirement for modeling polymicrobial infections in physiologically relevant conditions [65]. A key advantage of OoCs is real-time monitoring of tumor-microbiome interactions under dynamic conditions. Integrated sensors and transparent materials enable tracking of immune cell infiltration, tumor growth, and metabolic changes [66]. Platforms incorporating transepithelial/transendothelial electrical resistance or electrochemical sensors further enable assessment of barrier integrity in epithelial and endothelial tissues [67]. Furthermore, the incorporation of omics technologies into OoC platforms will enable molecular-level analysis of protein and gene expression, thereby deepening the understanding of disease progression and facilitating drug development [68,69]. Continued advancements including the integration of automated workflows, mathematical modelling, AI-assisted data analysis, and incorporation of patient-derived cells are progressively transforming these platforms into scalable, high-throughput systems with strong potential for precision medicine applications [70].

Despite their utility, existing tumor models remain constrained in their ability to precisely control physical, spatial, and biochemical microenvironmental parameters that critically influence microbial behavior [13]. In particular, most platforms lack tunable ECM properties, controllable diffusion gradients, and modular integration of multiple cell types. These limitations have motivated the development of biomaterial-based systems, which provide an engineering framework for reconstructing key physical, biochemical, and cellular features of the tumor microenvironments with greater control. The key advantages, limitations, and potential applications of the individual approaches are highlighted in Table 1.

**Table 1:** A summarizing table comparing 2D and 3D system models, along with their key features and limitations, to understand the influence of the microbiome in tumor models.

Model system	2D-culture	Spheroids	Organoids	Organ-on-a-chip (OOC)
**Tumor Microenvironment (TME) Fidelity**	● Low ● lacks native 3D architecture ● cell–ECM interactions	● Moderate ● improved cell–cell interactions ● gradient formation	● High ● recapitulates tissue-specific architecture ● functionality	● Very high ● dynamic microenvironment with fluidic ● mechanical cues

**Microbial Control**	● High ● easy to maintain sterile conditions	● Moderate	● Moderate to low	● Moderate

**Duration of microbiome co-cultures**	● Short-term	● Short/mid-term	● Short/mid-term	● Short/mid-term

**Scalability/Throughput**	● Very high	● Moderate to high	● Moderate	● Low to moderate

**Suitability/best used for**	● Suitable for preliminary drug screening ● mechanistic studies	● Useful for drug penetration ● tumor/stem cell studies ● Enables mechanistic studies	● Suitable for disease modeling personalized medicine ● Host-microbe interaction	● Highly suitable for therapeutic testing ● disease modeling

**Major Advantages**	● Simple ● cost-effective ● reproducible ● Controllable conditions ● Easily available for genetic engineering	● Mimics hypoxia ● nutrient gradients	● High physiological relevance ● self-organization ● tissue stiffness ● support both aerobic and anerobic bacteria ● Heterogenous cell population	● Mimics *in vivo* physiology ● tissue interfaces ● tunable stiffness ● Concentration gradients

**Key Limitations**	● Poor physiological relevance ● Lacks ECM ● rapid phenotypic loss	● Limited structural complexity ● reproducibility	● Batch variability ● difficult long-term maintenance ● Expensive growth factors	● Complex fabrication ● Expensive ● limited scalability ● Lack of cell heterogenicity and standardization

## 4 Biomaterials as platforms to model complex TME

Biomaterials provide a versatile platform for reconstructing key features of the *in vivo* TME that cannot be effectively modeled using conventional 2D dimensional cultures, spheroid systems alone or non-biological matrices [71]. While purely synthetic materials can support tumor cell growth, they often lack biologically relevant cues such as integrin-binding motifs present in the natural ECM components like collagen and gelatin. Consequently, these materials fail to fully capture the dynamic cell–matrix interactions that regulate tumor behavior in the TME. Moreover, both cancer cells and surrounding stromal populations are highly sensitive to mechanical properties of their microenvironment. Tumors respond to mechanical cues by actively remodeling their ECM, altering tissue stiffness and architecture during progression [72]. Conventional 2D culture systems are unable to model the diverse stiffness ranges present across tissues or capture how cells respond to mechanical perturbations. Similarly, critical processes such as tumor-driven angiogenesis remain poorly represented in simplified culture systems. Embedding cancer cells in 3D biomaterial matrices enable more accurate modeling of cell–matrix interactions, mechanical heterogeneity, and vascularization processes that collectively shape tumor progression.

In bacteria-integrated biomaterial cancer models (BIBCM), biomaterials serve additional functions beyond supporting tumor growth and stromal organization. These systems must simultaneously accommodate microbial viability, bacterial transport, and metabolite exchange while maintaining mammalian cell compatibility and spatial control over host–microbe interactions. Consequently, biomaterial selection in tumor–bacteria co-culture systems require consideration of parameters such as oxygen diffusion, matrix porosity, nutrient retention, bacterial permissiveness, immune compatibility, and prevention of uncontrolled microbial overgrowth or contamination.

### 4.1 Types of biomaterials and their advantages

Biomaterials employed in TME modeling can be broadly classified into natural ECM derived materials, synthetic polymers, hybrid or composite biomaterials, and tissue-derived decellularized scaffolds (Table 2). In BIBCM, biomaterial selection represents a critical design parameter because bacterial survival, metabolism, and spatial colonization are strongly influenced by matrix composition and transport properties. Consequently, biomaterial selection for BIBCM should consider not only conventional TME parameters such as stiffness, degradability, and biochemical signaling, but also bacterial compatibility criteria, including microbial viability, oxygen and nutrient diffusion, metabolite retention, microbial confinement, biofilm susceptibility, and immune compatibility. Different biomaterial classes exhibit distinct physicochemical characteristics that regulate these processes, thereby influencing tumor–microbe interactions and co-culture stability. Table 2 summarizes key biomaterial properties relevant to the construction of BIBCM platforms and highlights their respective advantages and limitations for modeling tumor–microbe interactions.

**Table 2:** Physicochemical and biological considerations of biomaterials used in bacteria-integrated biomaterial cancer models (BIBCM).

Biomaterial Class	Natural Derived	Synthetic Polymers	Hybrid or Composite	Tissue Derived
Representative Materials	Collagen, gelatin, Matrigel, hyaluronic acid, alginate	PEG, PLGA, PCL	PEG-collagen, GelMA-HAMA	Decellularized tumor or organ ECM

Key Physicochemical Properties	Hydrated structure, bioactive motifs, high permeability, enzymatically degradable	Tunable stiffness, pore size, degradation kinetics, defined chemistry	Combined bioactivity and tunability; spatial heterogeneity	Native ECM ultrastructure, tissue-specific composition

Potential Effects on Bacterial Survival	Supports bacterial adhesion, nutrient diffusion, metabolite retention, and microbial persistence; may facilitate biofilm formation	Lower intrinsic bacterial adhesion; controlled diffusion and microbial confinement; adjustable oxygen transport	Enables simultaneous support of tumor cells and controlled microbial colonization	Tissue-specific bacterial adhesion, metabolite diffusion, and microbial niche formation

Advantages for BIBCM	Physiologically relevant ECM signaling, supports tumor–microbe interactions and hypoxic gradients	High reproducibility, controllable bacterial localization, customizable transport properties	Balances microbial permissiveness with mechanical control; supports complex co-culture systems	Highly physiologically relevant architecture; useful for organ-specific tumor microbiome studies

Limitations	Batch variability, limited tunability, risk of bacterial overgrowth, poor reproducibility	Lack intrinsic bioactivity; may require functionalization for mammalian cell compatibility	More complex fabrication and characterization	Limited availability, donor variability, residual immunogenicity, reduced tunability

**Natural.** Natural ECM [73,74] derived materials including collagen [75,76], alginate [77], gelatin [[78], [79], [80]], hyaluronic acid [81,82], and Matrigel [75] provide inherent biochemical cues such as integrin-binding motifs and support cell adhesion, proliferation, and angiogenesis. Their hydrated and porous architecture may additionally facilitate bacterial infiltration, nutrient exchange, and retention of microbial metabolites, potentially supporting bacterial persistence within tumor-like niches. However, the biological complexity and degradability of natural matrices may also increase susceptibility to uncontrolled bacterial overgrowth and biofilm formation. These materials bring biologically relevant compositions which enable faithful recapitulation of cell–matrix interactions but have batch-to-batch variability causing reproducibility issues and limited tunability of mechanical properties.

**Synthetic.** On the other hand, synthetic polymers such as polyethylene glycol (PEG), poly(lactic-co-glycolic acid) (PLGA), and polycaprolactone (PCL) offer highly controllable mechanical properties, reproducibility, and tunable degradation profiles while they lack intrinsic bioactive motifs [83]. Compared to natural biomaterials, synthetic polymers generally exhibit lower intrinsic bacterial adhesiveness and reduced nonspecific microbial colonization due to their defined chemical composition and limited bioactive motifs. Their tunable pore size, diffusion characteristics, and degradation kinetics may enable tighter regulation of bacterial localization, oxygen transport, and metabolite exchange within tumor–bacteria co-culture systems [83,84].

**Hybrid or composite.** Hybrid or composite biomaterials such as PEG-collagen hydrogels or gelatin-hyaluronic acid bring together biological activity and flexible tunability between natural ECM derived biomaterials and synthetic polymers, respectively [12]. Importantly, hybrid systems may permit simultaneous optimization of mammalian cell compatibility and microbial control by integrating biologically permissive ECM components with synthetic regions that regulate bacterial diffusion and confinement. Such spatial tunability may be particularly valuable for modeling localized bacterial colonization observed in hypoxic or necrotic tumor regions.

**Tissue-derived.** Finally, tissue-derived decellularized scaffolds involve the removal of cellular components from native tissues or organs while preserving 3D structure and tissue-specific biochemical cues. The decellularized scaffolds provide an architecture that is physiologically relevant that guide tumor cell behavior and remodeling. Preservation of native ECM ultrastructure and tissue-specific biochemical composition may additionally influence bacterial tropism, microbial adhesion, and metabolite diffusion in a tissue-dependent manner. However, residual biological complexity and limited tunability may complicate control over bacterial growth dynamics and reproducibility across experiments. However, acquisition of tissues is limited due to potential immunogenicity, and reduced control over mechanical properties once decellularized.

Overall, the selection of a biomaterial platform depends on the specific TME features that are being modeled, including mechanical stiffness, ECM composition, and cell–matrix interactions. In the context of bacteria-integrated tumor models, these biomaterial classes also differ substantially in their capacity to support microbial survival and host–microbe interactions. Natural ECM-derived matrices may better support bacterial colonization, persistence, and metabolite retention due to their hydrated structure, intrinsic bioactivity, and enhanced permeability, whereas synthetic polymers offer greater control over pore architecture, diffusion characteristics, and bacterial confinement. Hybrid biomaterials may be particularly advantageous for tumor–bacteria co-culture because they enable simultaneous tuning of mammalian cell adhesion, microbial permissiveness, oxygen transport, and nutrient diffusion. Importantly, biomaterial platforms for bacterial integration must balance support of controlled microbial viability with prevention of excessive bacterial overgrowth or biofilm-associated contamination. The following sections describe how these platforms have been employed to study tumor cell behavior, mechanobiology, and angiogenesis in physiologically relevant contexts.

### 4.2 Cell-ECM interactions using biomaterial TME models

Although tumor spheroids enable investigation of cell-cell interactions within a 3D context, they do not adequately capture interactions between tumor cells and the surrounding ECM. Biomaterial-based matrices provide a more physiologically relevant platform by incorporating biochemical and structural features that mimic native tissue environments. For example, Pradhan et al. investigated disseminated tumor cell colonization using a set of 16 gels with tunable adhesion ligand density, enzymatic degradability, and mechanical properties [85]. Their findings demonstrated that adhesion ligand density and matrix degradability significantly influence the ability of disseminating tumor cells to transition into a proliferative state, highlighting the importance of ECM properties in regulating metastatic outgrowth. In order to more closely mimic the *in vivo* TME and investigate the impact of organ-specific microenvironments on cancer progression, Rafaeva et al. utilized decellularized mouse organs as biomaterial scaffolds to investigate organ-specific influences on cancer progression [86]. This approach preserved native ECM architecture and embedded biochemical cues, enabling analysis of tumor cell behavior within tissue specific microenvironments. The study demonstrated that cancer cells readily remodel ECM to establish tumor niches and exhibited signaling profiles more closely resembling *in vivo* metastatic behavior compared to cells grown in 2D systems.

These matrix-dependent interactions are also highly relevant in bacteria-integrated tumor systems, where ECM composition and degradability may influence bacterial adhesion, microbial penetration, and formation of localized microbial niches within tumors. Matrix architecture additionally regulates diffusion of bacterial metabolites, toxins, and quorum-sensing molecules that can alter tumor and stromal cell behavior. Therefore, biomaterials capable of recapitulating both tumor ECM remodeling and microbial transport dynamics may provide important platforms for studying spatially resolved tumor–microbiome interactions.

### 4.3 Modeling TME stiffness using 3D matrices

Mechanical properties vary widely across tissues and play a critical role in tumor development and progression [87]. Conventional 2D culture systems are limited to rigid substrates that fail to reflect physiologically relevant stiffness ranges. Biomaterial-based platforms enable precise tuning of matrix stiffness to investigate how mechanical cues regulate tumor behavior. Levental et al. demonstrated that increasing stiffness in collagen gels through oxidase-mediated crosslinking enhanced breast cancer tumorigenesis by promoting focal adhesion formation and mechanotransduction signaling [88]. Similarly, Jiang et al. showed that matrix stiffness preferentially promoted osteosarcoma cell growth, while adhesion ligand density more strongly influenced differentiation of non-cancerous osteoblasts [89]. In parallel, tunable hybrid hydrogel systems incorporating components such as hyaluronic acid methacrylate (HAMA) and gelatin methacrylate (GelMA) have been developed to model stiffness-dependent tumor behavior within multicellular spheroid systems [90]. Using this approach, hydrogels spanning physiologically relevant stiffness ranges were shown to regulate pancreatic cancer spheroid growth, morphology, and cell–matrix interactions, recapitulating key features of the desmoplastic tumor microenvironment. Advanced biomaterial designs have further enabled modeling of spatial stiffness heterogeneity within the TME. Peela et al. developed multi-stiffness hydrogels that stimulate adjacent tissues with differing mechanical properties [91]. Using this system, they showed that breast cancer cell migration patterns including directionality and persistence, were strongly influenced by local stiffness gradients, suggesting potential mechanisms underlying invasive behavior. Specifically, the authors noted that cells migrating through stiff hydrogels seemed to leave tracks behind through which subsequent cells could migrate, imparting a direction to the migration of cells from stiff matrices that were not present in cells migrating from soft matrices. Matrix stiffness also influences tumor–stroma interactions. Yue et al. reported that stromal cell differentiation into adipocytes was modulated by the stiffness of biomaterial matrices when co-cultured with breast cancer spheroids, highlighting the role of mechanical cues in regulating cellular crosstalk within the TME [92].

In BIBCM, matrix stiffness may additionally influence bacterial infiltration, motility, and localization within tumor tissues. Increased matrix density and reduced pore size can alter microbial transport and oxygen diffusion, potentially contributing to hypoxic microniches that favor growth of anaerobic or facultative anaerobic bacterial species frequently associated with tumors. Mechanical properties may also affect immune cell migration and bacterial clearance, highlighting the importance of integrating mechanobiology with host–microbe interactions in engineered tumor systems.

### 4.4 Modeling of angiogenesis in the TME

A tumor's ability to induce angiogenesis to sustain its metabolic needs is crucial to its survival, but modeling of new blood vessel formation is extremely limited in 2D culture tumor survival and progression. Culturing cells in 3D gels can allow cells to generate 3D tissue structures, including blood vessels. An early study investigating the capabilities of collagen matrices as a 3D growth environment demonstrated that endothelial cells encapsulated within collagen matrices could form vascular-like structures *in vitro* [93]. Subsequent work by Dr. Fischbach and colleagues expanded on these capabilities by co-culturing tumor cells with endothelial populations within biomaterial-based environments [94]. For example, Fischbach et al. showed that cancer cells in 3D biomaterial matrices comprised of alginate decorated with integrin-binding motifs exhibited increased expression of the pro-angiogenic cytokine IL-8 compared to 2D conditions [94]. Cross et al. further demonstrated that tumor cells embedded within collagen gels stimulated human umbilical vein endothelial cells (HUVECs) to invade the bulk gel from a monolayer, mimicking tumor angiogenesis processes *in vivo* [95]. They attributed this invasion to the known induction of expression of pro-angiogenic factors, including IL-8, due to culture in a 3D environment, which may induce hypoxia within the gel due to the density of the collagen gel [95,96]. In another study, Seano et al. were able to use tumor spheroids to induce angiogenesis in their *in vitro* angiogenesis model consisting of arterial explants cultured in Matrigel (Fig. 3) [34]. These spheroids were able to induce this angiogenesis in the absence of exogenous angiogenic factors, implying that the spheroids themselves were able to produce a strong enough pro-angiogenic signal to stimulate neoangiogenesis. These angiogenic and vascularized biomaterial systems may be particularly important for bacteria-integrated tumor models because vascular architecture strongly influences oxygen gradients, immune cell trafficking, and microbial dissemination within tumors. Since many tumor-associated bacterial species preferentially localize to hypoxic or necrotic regions, biomaterials capable of reproducing physiologically relevant oxygen and nutrient gradients may enable more accurate investigation of bacterial colonization dynamics and therapeutic responses.

Collectively, these studies demonstrate that 3D biomaterial models of the TME provide substantially greater capability to recapitulate *in vivo* tumor complexity than 2D or biomaterial-free systems by enabling control over cell–matrix interactions, tissue mechanics, angiogenesis, and spatial organization. For BIBCM specifically, biomaterials additionally serve as critical regulators of microbial viability, bacterial confinement, oxygen and nutrient diffusion, metabolite retention, and immune accessibility. The ability to tune matrix porosity, degradability, and biochemical composition positions biomaterials as uniquely powerful tools for engineering-controlled tumor–microbiome co-culture systems. By supporting simultaneous multicellular and multi-kingdom interactions, these platforms offer important opportunities to investigate microbial contributions to tumor progression, therapeutic response, and cancer-associated immune regulation.

## 5 Technologies and design principles for bacteria-integrated next-generation TME models

While biomaterial platforms enable reconstruction of key structural and mechanical features of the TME, the integration of microbial components introduces additional layers of complexity that require new engineering strategies. The recognition that bacteria actively colonize tumors and participate in diverse TME interactions has redefined the requirements for physiologically relevant TME models, shifting the focus from purely cellular systems to multi-kingdom environments [97]. Biomaterials play a central role in enabling these next-generation models by providing spatial control, microenvironmental regulation, and physical scaffolding necessary to support stable bacteria-tumor cocultures. This section highlights design principles and emerging technologies for constructing biomaterial-enabled bacteria-integrated TME models. This includes programmable microbial systems, mechanobiological interactions, metabolic and immune consequences, and engineering challenges associated with sustaining physiologically relevant cocultures.

### 5.1 Engineering bacteria-integrated biomaterial TME models

**Fig. 4:**
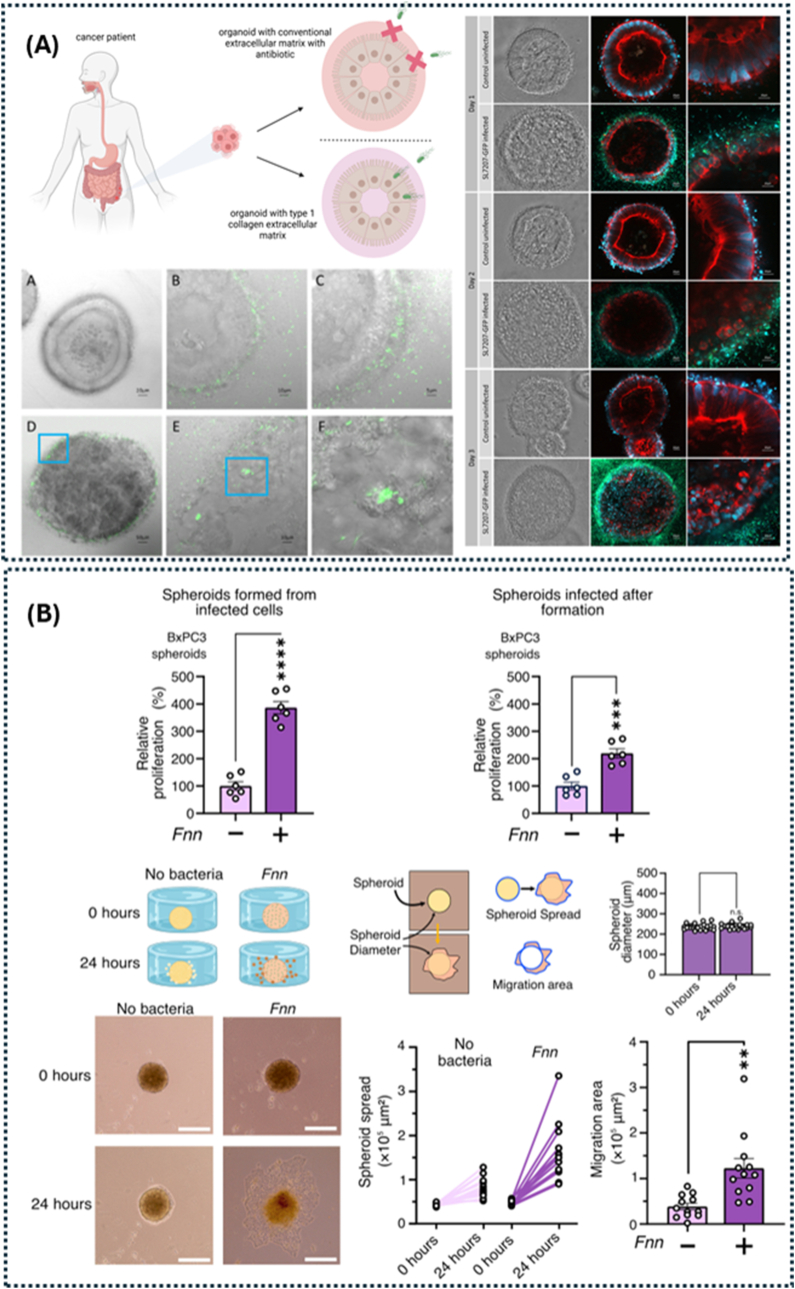
Examples of biomaterial-based TME modelling with integration of bacteria. **(A)** Collagen I-based scaffolds used in 3D tumor culture systems compatible with bacterial incorporation and co-culture [75]. (Graphical abstract, Fig. 5, Fig. 6 reproduced from Farrell, *Cells*, 2025, licensed under CC BY 4.0 (https://creativecommons.org/licenses/by/4.0/)) **(B)** Matrigel encapsulated tumor spheroids cultured in the presence or absence of *F. nucleatum*. Representative differences in spheroid morphology and spreading are shown [102]. (Fig. 6 reproduced from Udayasuryan et al., *Sci Signal*, 2022).

Designing bacteria-integrated TME models introduces a different set of constraints compared to conventional biomaterial model systems [10,16,98,99]. These platforms must simultaneously preserve tumor architecture, support bacterial viability, and enable controlled, measurable host–microbe interactions. Increasing system complexity (through microfluidics, multicellular co-culture, or microbial incorporation) improves biological relevance but reduces scalability and consistency, whereas simplified systems are more reproducible but fail to capture key tumor-microbiome dynamics [100,101]. The most effective systems will include matrix composition, oxygen gradients, bacterial strain, and immune inclusion, so that multi-kingdom interactions can be integrated systematically (Fig. 4) [75,102,103]. Firstly, ECM selection determines whether bacterial behavior will be observable. Collagen I has consistently emerged as a bacteria-permissive scaffold that supports invasion, persistence, and tumor interaction in both spheroids and organoids. The fibrillar, porous architecture of type I collagen supports bacterial penetration and direct host-cell contact. It also presents collagen-binding motifs recognized by adhesins across multiple bacterial species [104]. Matrigel, by contrast, is a basement membrane extract of heterogeneous composition. Its denser matrix impedes bacterial diffusion and limits viability within the scaffold. This was clearly demonstrated by Farrell et al. where replacing Matrigel with type I collagen enabled efficient colonization of CRC organoids by *Salmonella* SL7207, with significantly higher infection efficiency and sustained expansion [75]. Within these collagen-based systems, bacterial populations increased more than tenfold over four days and gradually penetrated patient-derived organoid structures, demonstrating active bacterial colonization and growth within biomaterial-supported tumor models [75].

Bacterial proliferation within biomaterial systems is influenced by matrix mechanics and spatial confinement. Colony expansion rates decrease as matrix stiffness increases, suggesting that surrounding materials can affect bacterial growth behavior [105,106]. As a result, biomaterials used in bacteria-integrated systems need to maintain sufficient stiffness and toughness while still allowing bacterial survival and expansion. Hydrogels composed of polyvinyl alcohol (PVA) combined with alginate [107], gelatin [108], or agarose [109] can provide mechanical stability together with high fatigue resistance and support bacterial viability. Recent implantable living material (ILM) systems further demonstrated that engineered *E. coli* can remain viable within PVA-based scaffolds for extended periods while retaining genetic sensing functionality, illustrating how biomaterials can support durable bacterial persistence and circuit stability [110]. Beyond matrix mechanics, bacterial colonization within tumor models is also influenced by bacterial motility and tumor-associated metabolic gradients. Tumor-associated metabolic gradients and hypoxic niches can further promote bacterial accumulation and persistence within biomaterial-supported tumor systems. Many bacteria accumulate in tumors through chemotactic responses toward TME-associated metabolites such as lactate, while anaerobic metabolic programs further favor bacterial persistence within hypoxic and necrotic tumor regions [111]. These properties allow bacteria to establish localized proliferative niches within biomaterial-supported tumor systems.

Matrix architecture can also be leveraged to spatially control bacterial populations. Porous scaffolds (e.g., alginate or polymer composites) create localized microhabitats that support bacteria without overwhelming tumor cells, while stimuli-responsive hydrogels enable dynamic control of bacterial growth through environmental cues such as pH or metabolite accumulation [77]. These approaches allow bacterial expansion to be regulated without disrupting overall tumor structure. Equally important is bacterial strain selection and mode of incorporation, which define what can be measured in the system. Tumor-targeting strains such as attenuated *Salmonella* are well suited for studying colonization dynamics within defined ECM environments [75]. In contrast, tumor-associated anaerobes such as *F*. *nucleatum* are often used in collagen-embedded spheroid systems to isolate tumor-intrinsic responses. For example, infection of pancreatic cancer spheroids with *F. nucleatum*, followed by embedding in collagen I, resulted in enhanced tumor cell migration and increased secretion of cytokines including GM-CSF, CXCL1, and IL-8 [18,112]. These simplified systems allowed for clearer interpretation of bacteria-driven phenotypes. The most reproducible models in this space share a few common features: defined bacterial inputs (strain and viability), controlled containment strategies to prevent overgrowth, and quantitative readouts of both microbial and tumor responses [10,16,45]. Effective modeling of engineered bacterial therapeutics requires capturing the full temporal life cycle of the vector, not only its initial localization. *In vivo*, bacterial vectors undergo sequential phases: tumor entry and colonization, local proliferation, plateau-phase payload production governed by quorum-sensing circuits, and eventual clearance through nutrient limitation, matrix restriction, antibiotic intervention, or immune attack [113,114]. Quorum-regulated lysis circuits exemplify this temporal complexity: bacteria can be programmed to release therapeutic cargo at a population density threshold and reseed through surviving cells, generating pulsatile delivery profiles [115,116]. Biomaterial matrices can be designed to recapitulate these phases by modulating pore size, crosslinking density, degradation rate, oxygen diffusivity, and nutrient transport [117]. Permissive matrices favor bacterial expansion and persistence; denser or stimuli-responsive scaffolds restrict bacterial spread and gate payload release. Bacteria-integrated biomaterial models, therefore, allow quantification of colonization kinetics, persistence duration, payload production timing, and how clearance mechanisms remodel the TME over time. Despite this progress, most current systems still capture only a subset of TME features, typically hypoxia and bacterial localization, while immune, stromal, and vascular components remain difficult to integrate alongside viable microbes [10,73,98,118].

**Fig. 5:**
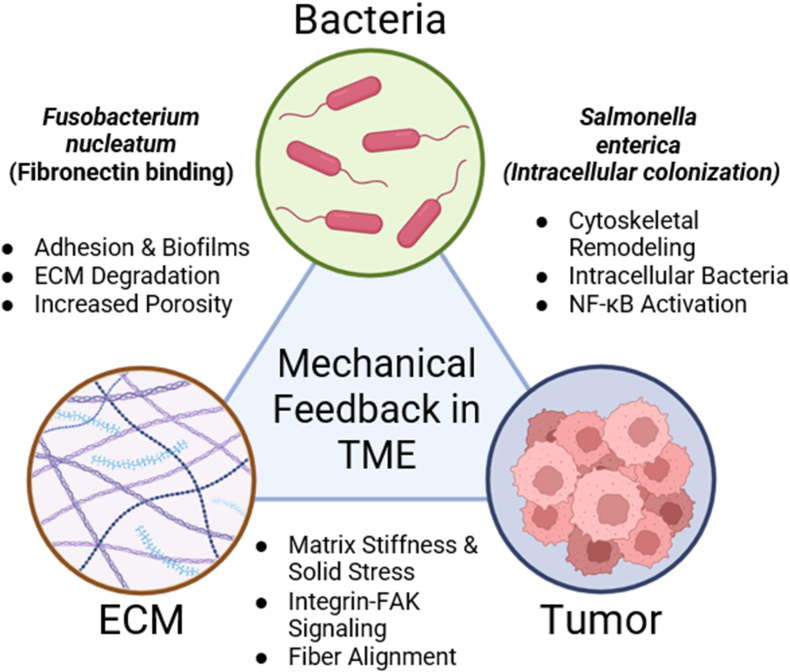
Proposed mechanobiological interactions among bacteria, tumor cells, and the extracellular matrix (ECM). Schematic illustration summarizing reported and proposed interactions between bacteria, tumor cells, and the ECM within the TME. Potential processes include bacterial adhesion, extracellular matrix remodeling, enzymatic degradation, and biofilm formation, which may influence matrix organization and mechanical properties. Tumor cells interact with the ECM through integrin-associated mechanotransduction and cytoskeletal remodeling. The figure summarizes emerging concepts regarding bacteria-associated biomechanical interactions in tumor-associated environments.

**Fig. 6:**
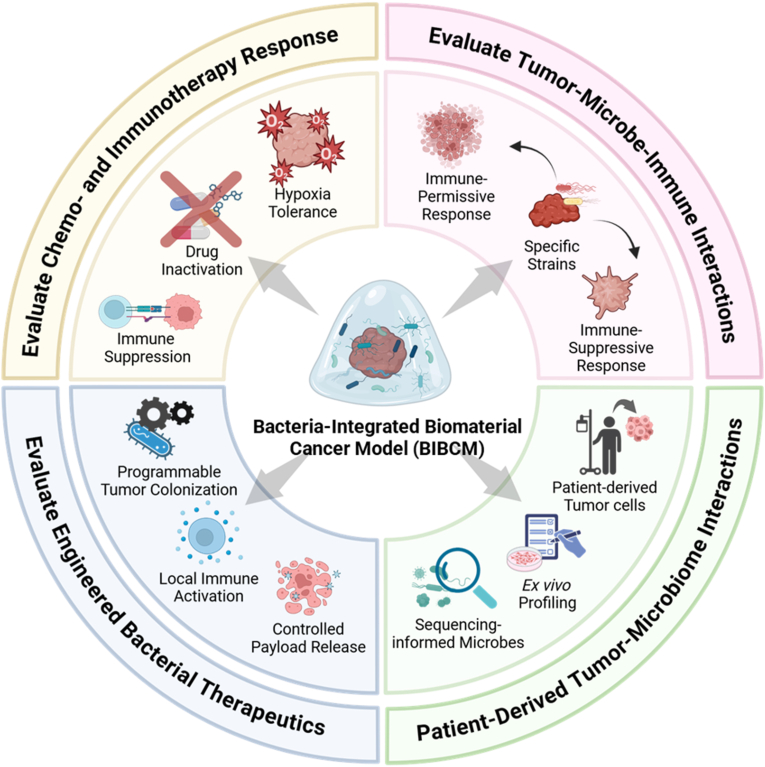
Potential applications of bacteria-integrated biomaterial cancer models (BIBCM). Schematic overview of 3D platform combining tumor cells, bacterial populations, and, where relevant, immune components to study tumor-microbiome interactions under controlled experimental conditions. Representative applications of BIBCM include the evaluation of chemo- and immunotherapy response (yellow) driven by microbial metabolism, immune modulation, and hypoxia, evaluation of engineered bacterial therapeutics (blue) for targeted payload delivery and immune activation, evaluation of tumor-microbe-immune interactions (red) using defined strains, consortia, and metabolites, and understanding patient-derived tumor-microbiome interactions (green) with patient-derived tumors and matched microbial communities for *ex vivo* drug profiling. Together, these platforms enable integrated microbial interrogation and therapeutic screening. (For interpretation of the references to color in this figure legend, the reader is referred to the Web version of this article.)

### 5.2 Mechanobiology of bacteria–tumor and extracellular matrix (ECM) interactions

In the TME, mechanobiology is a well-established regulatory axis governing tumor ECM interactions but remains an emerging and largely unverified concept in bacteria–ECM and bacteria–tumor contexts (Fig. 5). In tumors, mechanical cues such as matrix stiffness, confinement, and shear stress are known to regulate cancer cell behavior through mechanotransduction pathways, including integrin signaling and YAP/TAZ activation [13,72]. By contrast, bacterial mechanosensing has been primarily studied in non-tumor contexts, including surface attachment, biofilm formation, and envelope stress responses during infection or on abiotic substrates. Whether these mechanobiological processes interact meaningfully in tumor-associated microbial niches remains an open question.

**Tumor cell mechanotransduction in ECM.** Cancer cells sense and respond to mechanical cues such as environment for stiffness or pressure, although through distinct mechanisms [[119], [120], [121], [122], [123], [124]]. Tumor cells convert physical stimuli as matrix stiffness, confinement, and mechanical stress into biochemical signals for gene expressions which result in mechanoresponsive, resulting changes in the cells. In particular, integrin-mediated adhesion complexes and downstream signaling pathways such as YAP/TAZ play central roles in coupling ECM mechanics to tumor progression [72,125]. Importantly, this mechanobiological framework is well established in tumor biology, where ECM remodeling and mechanical heterogeneity are recognized hallmarks of disease progression.

**Bacterial mechanosensing in non-tumor systems and with ECM.** Bacteria similarly respond to mechanical and matrix-associated cues through adhesion, motility modulation, and quorum sensing which influence surface attachment, colonization, and biofilm formation with ECM components [123,124,126,127]. While bacterial mechanosensing has been extensively studied in abiotic and infection contexts, its role within tumor tissues remains unexplored. Particularly, ECM structural heterogeneity and matrix remodeling shape the biochemical and mechanical signals experienced by both tumor cells and intratumoral bacteria [[127], [128], [129]]. Emerging evidence also suggests that bacteria can both respond to and remodel ECM components within tumor-associated environments [97]. Recent reviews describe how intratumoral bacteria localize within collagen-rich and fibrotic niches, where ECM composition, porosity, and stiffness may regulate bacterial adhesion, persistence, and spatial distribution within tumors [[127], [128], [129]]. Conversely, bacterial activities including biofilm formation, protease secretion, and modulation of matrix-degrading enzymes may alter local ECM architecture and tissue mechanics, potentially establishing reciprocal biomechanical interactions between microbes and tumor tissues [[127], [128], [129]]. Although these observations remain largely correlative and mechanistic studies are still limited, they support the concept that bacteria are not only passive occupants of the TME but may actively participate in ECM-associated remodeling processes.

**Mechanical interactions between tumors and bacteria in the TME.** Although direct studies in tumor-bacteria co-culture systems remain limited, several findings suggest that ECM mechanics may indirectly influence microbial behavior in tumor-like environments. Matrix mechanics influences bacterial localization, invasion modes, persistence, and enzymatic activity, creating bidirectional mechanical feedback between tumor cells and microbes [130]. Studies have shown that engineered ECM stiffness alters bacterial invasion patterns and antibiotic efficacy, linking matrix mechanics to host–bacteria crosstalk [122,[130], [131], [132]]. Aparicio-Yuste et al. also demonstrated that there is mechanical competition between infected and uninfected epithelial cells and constrains bacterial spread, combining computation and experiments on defined stiffness substrates [131,133]. These observations suggest that mechanical properties of the ECM could, in principle, influence bacterial localization and persistence in tumor-associated environments; however, such interactions remain largely inferred rather than directly demonstrated in tumor models. The addition of bacteria could turn the same mechanical signals (stiffness, traction, confinement) into multi-kingdom regulatory cues for cancer cells as Wullkopf have shown cancer cells’ capability to adapt to varying stiffness which also contains bacteria [121,134].

Current evidence supports a clear distinction between well-established tumor mechanobiology and emerging, indirect evidence for bacterial involvement in mechanically regulated microenvironments. Within this framework, the complex mechanobiology between the bacteria and ECM as well as between tumor and ECM poses mechanobiology as a design parameter needs to be considered when modeling TME. 3D biomaterial matrices with tunable stiffness, viscoelasticity, and architectural complexity are also required to investigate how mechanical cues regulate microbial colonization and tumor progression within integrated systems.

### 5.3 Metabolic crosstalk/immune consequences

Intratumoral bacteria influence tumor behavior primarily through metabolic activity and immune modulation, making these two axes essential design considerations in bacteria-integrated tumor models [31,135]. Unlike purely mammalian systems, microbial incorporation introduces additional layers of complexity, including nutrient competition, metabolite accumulation, and immune signaling that are highly dependent on spatial context. One of the primary challenges is controlling local metabolite concentrations. Bacteria consume key nutrients such as glucose and amino acids while producing bioactive metabolites, including short-chain fatty acids (SCFAs), bile acid derivatives, and tryptophan metabolites [[136], [137], [138]]. These effects are not uniform and depend on local gradients and confinement. As a result, models that do not control diffusion or spatial organization often fail to capture physiologically relevant microbial effects [71,73,[87], [88], [89],^99^]. For example, butyrate production can either suppress tumor growth or promote invasiveness and immunosuppression depending on its local concentration and the surrounding cellular context, highlighting the importance of controlling metabolite exposure within the system [137]. Matrix design therefore becomes a critical regulator of metabolic signaling. Parameters such as pore size, crosslinking density, and network structure directly determine diffusion of microbial metabolites and nutrient availability. Systems that allow unrestricted diffusion can dilute microbial effects, while overly restrictive matrices may prevent meaningful host–microbe interaction. The goal is to engineer matrices that enable localized metabolite accumulation without global toxicity or nutrient depletion, maintaining both bacterial viability and tumor cell function.

Following bacterial colonization and proliferation within tumors, anti-tumor immune responses develop progressively through interactions among bacterial populations, tumor cells, and immune components. Bacteria stimulate immune signaling through pathogen-associated molecular patterns (PAMPs), including CpG-rich DNA, flagellin, and lipopolysaccharide, which activate innate immune pathways and promote pro-inflammatory cytokine production, dendritic cell maturation, and recruitment of cytotoxic immune populations [[139], [140], [141], [142]]. Engineered bacteria may further enhance these responses through localized secretion of immunostimulatory payloads, helping overcome local immune suppression and improve therapeutic efficacy. In parallel, several bacterial species can directly recruit inflammatory and cytotoxic immune cells into the TME, contributing to tumor clearance [143,144]. Together, these findings highlight that bacteria-based cancer therapies evolve through dynamic and time-dependent phases of colonization, persistence, immune activation, and eventual immune-mediated clearance.

Modeling these immune-related processes within bacteria-integrated biomaterial tumor systems remains challenging. Many clinically relevant tumor-microbiome interactions are immune-mediated, yet most current models either exclude immune cells or fail to maintain stable co-culture in the presence of bacteria [6,10,16,19,20,26,45,145,146]. This creates a key gap between short-term mechanistic systems and physiologically relevant models capable of capturing longitudinal bacterial persistence and clearance dynamics. For example, bacterial metabolites and signaling pathways can drive macrophage polarization, dendritic cell activation, and cytokine production, but these effects are highly dependent on timing, spatial proximity, and microbial load. Without control over these variables, immune responses can become either exaggerated or suppressed in ways that are not biologically meaningful. Several studies illustrate how simplified systems can isolate these effects while also highlighting current limitations. Incorporation of *F. nucleatum* into tumor spheroids has been used to demonstrate increased pro-inflammatory cytokine signaling, including IL-8, in the absence of immune cells, enabling clearer interpretation of tumor-intrinsic responses [18,41]. Similarly, microbiome-derived metabolites such as butyrate have been shown to alter macrophage polarization and tumor invasiveness in controlled 3D systems. Overall, integrating metabolic and immune components into next-generation bacteria-integrated TME models requires a controlled metabolite gradients, stable co-culture of microbial and immune populations, and quantifiable readouts of both tumor and immune responses.

Temporal control is particularly important when immune components are incorporated, because immune-cell recruitment may progressively shift a bacteria-permissive model system toward immune-mediated bacterial clearance and tumor remodeling over time. Sequential sampling of bacterial burden, cytokine release, immune-cell activation, tumor viability, and metabolite accumulation can therefore distinguish persistent colonization from immune-mediated clearance [116,147]. This is especially relevant for engineered bacteria, where therapeutic activity may depend on maintaining an optimal window of bacterial viability and payload production without uncontrolled overgrowth or excessive inflammation [116,147]. Biomaterial platforms, particularly hydrogel-based systems, may help tune this balance by supporting nutrient diffusion and microbial activity while limiting premature loss of bacterial viability or uncontrolled dissemination [117].

## 6 Applications for bacteria-integrated biomaterial cancer models (BIBCM)

The integration of bacteria into biomaterial tumor models enables a shift from descriptive observations of tumor-associated microbes to controlled, mechanistic investigation of tumor–microbe interactions and how microbes shape tumor biology, including their emerging therapeutic exploitation within the TME [148]. The biomaterial properties summarized in Table 2 critically influence the ability of BIBCM platforms to model microbial metabolism, therapeutic response, immune signaling, and patient-specific tumor-microbiome interactions. This section highlights key applications where these platforms provide unique experimental advantages, particularly in dissecting therapeutic resistance, evaluating microbiome-informed interventions, and developing engineered or patient-specific systems.

### 6.1 Using BIBCM to evaluate chemo- and immunotherapy response

Therapeutic resistance has traditionally been conceptualized as a tumor-intrinsic process driven by acquired mutations, adaptive signaling rewiring, or epigenetic plasticity. However, growing experimental evidence indicates that components of the TME can materially influence treatment outcomes. Among these, tumor-associated bacteria have emerged as a potentially significant yet incompletely characterized contributor to therapeutic failure [1,135,149,150]. One of the most direct demonstrations of bacteria-associated resistance involves microbial drug metabolism [4,135]. Intratumoral *Gammaproteobacteria* expressing a long isoform of cytidine deaminase (CDD_L_) have been shown to convert gemcitabine into an inactive metabolite, thereby reducing chemotherapeutic efficacy in tumor models and providing a mechanistic basis for gemcitabine resistance in pancreatic ductal adenocarcinoma (PDAC) [4]. Notably, antibiotic-mediated depletion of these bacteria restored drug sensitivity in resistance, indicating that the resistance was not solely attributable to tumor cell genetics. Such findings illustrate how microbial enzymatic activity within the TME can modulate local drug bioavailability and complicate interpretation of intrinsic tumor sensitivity in sterile *in vitro* systems [1]. The ability of BIBCM platforms to model microbial drug metabolism and hypoxia-dependent therapeutic resistance is strongly influenced by biomaterial properties including oxygen diffusion, matrix permeability, and metabolite retention.

Microbial influences have also been implicated in modulating immune responses relevant to checkpoint blockade [20,135,145]. Variability in response to PD-1/PD-L1 inhibition has been associated with differences in microbiome composition [20,146]. While these studies primarily examined gut microbiota, they establish a principle that microbial signals can shape dendritic cell activation, cytokine production, and CD8^+^ T cell priming. By extension, tumor-resident bacteria may influence local immune architecture within the TME, potentially reinforcing immune-excluded niches or altering checkpoint signaling dynamics [1,151]. Incorporating defined bacterial populations into these matrices permits systematic evaluation of whether resistance to immune checkpoint inhibitors reflects tumor genetics, matrix-mediated immune exclusion, microbial immunomodulation, or their combined effects that are not captured in conventional sterile tumor models. Beyond direct drug metabolism and immune modulation, bacteria may also contribute to resistance through metabolic and physicochemical mechanisms. Microbial oxygen consumption can intensify intratumoral hypoxia, stabilizing hypoxia inducible factor(HIF)-dependent transcriptional programs associated with therapeutic tolerance [152]. Additionally, microbial metabolites, including SCFAs and tryptophan-derived compounds, have been shown to modulate immune cell function and redox balance. These findings suggest that resistance is not always driven by fixed tumor genetics but can also be sustained through metabolic interactions between tumor cells and resident bacteria [153]. Rather than replacing genetic models, this approach complements them by directly capturing microbial contributions to therapeutic responses.

### 6.2 Using BIBCM to evaluate engineered bacterial therapeutics

In addition to chemo- and immune-therapies, these platforms also provide a controlled environment to evaluate engineered bacterial therapeutics. Synthetic biology has enabled the development of bacteria as programmable therapeutic platforms capable of selectively colonizing tumors and delivering bioactive payloads [37]. Several bacterial genera, including *Escherichia, Salmonella, Listeria*, and *Clostridium*, exhibit intrinsic tumor tropism, particularly within hypoxic regions of the TME, making them attractive candidates for targeted therapy [1,4,30,151]. Biomaterial selection is particularly important for engineered bacterial therapeutics because matrix porosity, degradation kinetics, and bacterial confinement directly influence colonization stability and payload release dynamics. However, native bacterial colonization alone is insufficient for tumor clearance, necessitating genetic engineering approaches that enable controlled payload delivery, immune activation, and microenvironment remodeling. Engineered strains of *Escherichia coli* Nissle 1917 (EcN) represent one of the most advanced platforms in this space due to their safety profile, tumor-targeting capability, and genetic tractability [[154], [155], [156]]. EcN has been engineered to modulate the TME through both metabolic and immune mechanisms. For example, strains engineered to produce L-arginine enhance CD8^+^ T-cell activation and improve response to PD-L1 blockade, while others have been designed to secrete immune-recruiting proteins or bispecific T-cell engagers. Synthetic gene circuits, including quorum-sensing systems, enable spatially restricted activation of therapeutic payloads within tumors.

Similarly, attenuated *Salmonella enterica serovar Typhimurium* has been widely developed as a tumor-targeting vector due to its preferential accumulation in hypoxic tumor regions [[157], [158], [159]]. Engineered strains defective in ppGpp synthesis (ΔrelA/ΔspoT) retain tumor tropism while reducing pathogenicity and have been modified to deliver cytotoxic or immunostimulatory payloads, including flagellin B, cytolysin A, and cytokines such as IL-2, IL-15, and IFNγ. These systems demonstrate how engineered bacteria can simultaneously target tumor cells and activate immune responses within the TME. Other platforms, including engineered *Listeria monocytogenes*, have advanced into clinical evaluation [160,161]. These strains are designed to deliver tumor-associated antigens, such as mesothelin, while activating both innate and adaptive immune responses through Toll-like receptor (TLR) signaling. Early clinical studies have demonstrated their ability to induce tumor-specific T-cell responses, highlighting the translational potential of engineered bacterial therapies. Commensal species such as *Lactobacillus* have also been shown to suppress tumor growth through metabolite production, while engineered strains can deliver immunomodulatory molecules that enhance anti-tumor immunity [7,19,25,146].

Despite these advances, several challenges remain, including control of bacterial proliferation, safety concerns related to horizontal gene transfer, and variability in payload delivery. This is where BIBCM provide a critical advantage. These systems allow precise control over bacterial localization, growth dynamics, and microenvironmental context, enabling systematic evaluation of engineered strains under conditions that more closely resemble the TME. By tuning matrix properties and spatial confinement, these models can be used to assess payload release kinetics, immune activation, and therapeutic efficacy while minimizing confounding variables.

### 6.3 Using BIBCM to evaluate tumor-microbe-immune interactions

Beyond therapeutic outcomes, these models enable mechanistic dissection of how intratumoral bacteria regulate immune cell function and tumor signaling through direct interactions and metabolite-mediated pathways. By incorporating defined microbial strains into structured 3D tumor systems, these models enable systematic evaluation of how specific bacteria influence immune activation, cytokine signaling, and therapeutic response under controlled architectural and diffusion conditions [20,145,146]. Several studies highlight the importance of strain-specific effects on therapy outcomes. For example, *Enterococcus* species enhance anti-PD-L1 efficacy through SagA-mediated activation of NOD2 signaling, while *Bifidobacterium* strains can improve checkpoint blockade via TLR2-dependent immune activation [162]. In contrast, tumor-associated taxa such as *F. nucleatum* are linked to poor immunotherapy response, particularly in colorectal and pancreatic cancers [18,30,41]. These observations suggest that microbial composition can shift tumors toward either immune-permissive or immune-suppressive states, but the underlying mechanisms remain difficult to isolate *in vivo* [31,135,163,164]. Bacteria-integrated tumor models allow these effects to be tested directly by controlling microbial composition, spatial distribution, and tumor architecture [43,60,71,73,75,98,99]. In these systems, biomaterial transport properties and spatial architecture critically regulate microbial metabolite gradients, immune cell infiltration, and localized cytokine signaling. More broadly, recent biomaterial-based cancer models and therapeutic platforms have highlighted the importance of engineered microenvironments for regulating immune signaling, drug delivery, and tumor responsiveness in translational cancer therapy [165]. For example, patient-derived organoids embedded in ECM-mimetic matrices can be exposed to defined microbial communities to assess changes in immune cell recruitment, epithelial barrier function, and cytokine signaling. This provides a functional framework to evaluate whether taxa identified in sequencing studies actively drive therapeutic response or simply correlate with it.

In addition to whole organisms, microbiome-derived metabolites represent another key axis of therapeutic modulation. SCFAs such as butyrate, produced by species including *Clostridium* and *Bifidobacterium*, have been shown to enhance CD8^+^ T-cell function and improve checkpoint blockade responses [19,136,137]. Similarly, tryptophan-derived metabolites can regulate T cell activity through aryl hydrocarbon receptor signaling, while tumor-driven tryptophan depletion (e.g., via IDO activity) contributes to immune suppression [19,138,150,152]. These pathways highlight a dynamic interplay between microbial metabolism and host immune regulation that is highly dependent on local concentration and spatial context. This reinforces the need for systems capable of controlling metabolite gradients and spatial compartmentalization, particularly as biomaterial-enabled delivery platforms―including exosome-inspired and nanomaterial-based systems―are increasingly explored to modulate immune signaling and therapeutic responses within the tumor microenvironment [[165], [166], [167]]. A key limitation of current BIBCM is their limited ability to model recruitment of peripheral immune cells into bacteria-colonized tumor sites. Integrating these platforms with perfusable microfluidic vascular networks or OoC systems could enable circulating T cell repertoires or myeloid cells to be introduced under flow, allowing quantitative analysis of endothelial adhesion, extravasation, and infiltration into tumor matrices in response to bacterial colonization-induced chemokine or cytokine gradients [10,168]. Overall, by understanding these interactions, these platforms can enable the identification of microbial strains, metabolites, and consortia that can be leveraged to enhance cancer treatment.

### 6.4 Using BIBCM to understand patient-derived tumor-microbiome interactions

Personalized cancer models have advanced significantly through patient-derived systems, including organoids, xenografts, and tumor cell clusters, which preserve tumor histology, genetic profiles, and, in some cases, immune context [43,46,47,50,169]. These platforms have improved the ability to predict therapeutic response by accounting for interpatient variability. However, they largely treat the TME as a mammalian system and do not incorporate patient-specific microbial components, which are increasingly recognized as active regulators of tumor behavior. Variability in clinical outcomes may, in part, reflect patient-specific tumor–bacteria interactions, which are not captured in current personalized models.

Despite this promise, integrating patient-derived microbiota into tumor models presents substantial technical and translational challenges. Microbial composition is highly sensitive to *ex vivo* handling, oxygen exposure, and culture conditions, making preservation of clinically relevant communities difficult. In addition, tumor biopsies often contain limited and spatially heterogeneous bacterial populations, while bacteria and mammalian cells require fundamentally different growth conditions with respect to oxygen tension, nutrients, and growth kinetics. These factors complicate the establishment of stable and reproducible co-culture systems and increase the risk of bacterial overgrowth or loss of microbial diversity. Preserving patient-derived microbial viability within *ex vivo* systems further require biomaterials capable of maintaining physiologically relevant oxygen gradients and nutrient exchange while limiting uncontrolled bacterial expansion. Further challenges include the lack of standardized definitions for “patient-matched” microbiota and the limited immune complexity of most patient-derived models, which restricts accurate recapitulation of microbiome-mediated immune interactions within the TME.

Bacteria-integrated biomaterial platforms provide a potential route to address some of these limitations by enabling controlled incorporation of microbial components into ECM-mimetic hydrogels and organoid-supporting matrices [39,71,78,79,82,86,92]. By incorporating either patient-derived bacteria or defined microbial consortia informed by sequencing data, these systems allow the understanding of how microbial composition influences tumor behavior, immune signaling, and therapeutic response within an individualized context. However, such approaches must carefully balance microbial viability with tumor stability and experimental reproducibility. Within these constraints, these platforms may enable mechanistic studies of how microbial composition influences tumor behavior, immune signaling, and therapeutic response in an individualized context. This approach also enables evaluation of microbiome-targeted therapeutic strategies alongside standard cancer treatments. For example, targeting *F. nucleatum* using antimicrobial or nanoparticle-based approaches has been shown to restore drug sensitivity and enhance immune activation in preclinical models [[170], [171], [172]]. Similarly, engineered probiotic systems including lysis-circuit bacteria delivering checkpoint inhibitors, illustrate how microbial populations can be actively manipulated to remodel the TME [171,173]. Embedding these strategies within patient-derived biomaterial models allows simultaneous assessment of tumor response, immune modulation, and microbial intervention within a single, controlled system. Integrating microbial components into patient-derived tumor models represents a critical step toward expanding precision oncology beyond tumor genomics to include the microbial dimension of the TME.

## 7 Conclusion and future directions

Tumors are increasingly recognized as complex ecological systems in which malignant, stromal, immune, and microbial components interact dynamically to shape disease progression and therapeutic response. Although sequencing and imaging studies have firmly established the presence of bacteria in diverse human tumors, mechanistic understanding of how these microorganisms influence tumor biology remains limited. A major challenge has been the lack of experimental platforms that incorporate microbial variables into physiologically relevant TME under controlled conditions.

Traditional *in vitro* systems, animal models, and patient-derived platforms have provided important insights into tumor–host interactions, but they offer limited control over the physical, spatial, and biochemical parameters that govern tumor–microbe crosstalk. Biomaterial-based systems help address this gap by enabling precise reconstruction of key TME features, including matrix mechanics, diffusion gradients, and multicellular organization. In this context, bacteria can be introduced as defined and experimentally tractable variables rather than treated as uncontrolled contaminants, allowing researchers to examine how microbial localization, abundance, metabolic activity, and host-cell interactions influence cancer progression and treatment response.

The integration of bacteria into tumor models has already begun to reveal previously underappreciated dimensions of tumor biology, including microbial contributions to therapeutic resistance, immune modulation, and response heterogeneity. However, several challenges must be addressed before these systems can achieve broad adoption and translational impact. Standardized methods for bacterial incorporation, viability assessment, spatial quantification, co-culture with mammalian cells, and contamination control will be essential for improving reproducibility across laboratories. In addition, incorporation of live or engineered bacterial strains introduces important biosafety and regulatory considerations, including containment, prevention of unintended microbial dissemination, and control of horizontal gene transfer. Future development of these systems will therefore require integration of biosafety engineering strategies, standardized regulatory frameworks, and reproducible quality control measures, alongside close collaboration across biomaterials science, microbiology, synthetic biology, systems immunology, and clinical oncology.

Future advances should initially focus on improving reproducibility and experimental control in bacteria-integrated tumor systems before incorporating increasing biological complexity. Building on these foundational systems, next-generation platforms may integrate polymicrobial communities, vascularized perfusion, functional immune compartments, and physiologically relevant oxygen and nutrient gradients. Integration with patient-derived organoids, primary stromal and immune cells, and longitudinal clinical or microbiome data may further enhance the predictive value of these systems for therapeutic modeling. Coupling these platforms with microbiome sequencing, multi-omics profiling, and synthetic biology approaches may also enable identification of microbial biomarkers associated with treatment response and support development of programmable bacterial therapeutics and personalized microbiome-informed therapeutic strategies. Furthermore, spatial transcriptomics and emerging spatial host–microbiome sequencing approaches can relate bacterial localization to tumor-cell states, stromal organization, and immune-cell programs within intact tissue architecture [[174], [175], [176]]. Complementary spatial metabolomics can further define local metabolic niches, including microbial metabolite accumulation, nutrient depletion, hypoxia-associated metabolic programs, and intratumoral drug distribution [177,178]. These *in situ* technologies provide a framework for testing whether engineered bacteria–material–tumor–immune interactions in BIBCM recapitulate clinically observed spatial patterns in patient biopsies, thereby strengthening their functional and translational validation. Beyond spatial validation, BIBCM may also provide a platform for optimizing engineered interactions among bacteria, mammalian cells, and biomaterial scaffolds [147,179]. In addition to engineering bacterial strains for tumor targeting or payload delivery, mammalian cells within these systems could be engineered or chemically primed to improve therapeutic gene-product expression after DNA- or mRNA-based delivery. Recent work showing that small-molecule treatment can enhance synthetic gene expression in mammalian cells highlights an additional design axis for tuning payload output within bacteria–mammalian cell–biomaterial systems [180]. Collectively, BIBCM provide a framework for studying tumors as multi-kingdom ecosystems with greater mechanistic and spatial precision. As these platforms become more standardized, scalable, and patient relevant, they have the potential to advance fundamental cancer biology, improve preclinical therapeutic testing, and support more personalized approaches to cancer therapy.

## CRediT authorship contribution statement

**Keuna Jeon:** Conceptualization, Visualization, Writing – original draft, Writing – review & editing. **Uijin Kim:** Writing – original draft, Writing – review & editing. **Chang-Hun Ji:** Writing – original draft, Writing – review & editing. **Meenakshi Kamaraj:** Writing – original draft, Writing – review & editing. **Noah Zachary Laird:** Writing – original draft, Writing – review & editing. **Zhikun Wang:** Writing – original draft. **Hongxiao Yu:** Writing – original draft. **Menekse Ermis:** Writing – original draft. **Ruby May A. Sullan:** Writing – review & editing. **Xiling Shen:** Writing – review & editing. **Natashya Falcone:** Conceptualization, Supervision, Writing – original draft, Writing – review & editing.

## Declaration of competing interest

The authors declare that they have no known competing financial interests or personal relationships that could have appeared to influence the work reported in this paper.

## Data Availability

No data was used for the research described in the article.

## References

[1] Nejman D. (2020). Science.

[2] Zhang Z. (2022). Eur. J. Immunol..

[3] Hou T. (2025). Adv. Drug Deliv. Rev..

[4] Geller L.T. (2017). Science.

[5] Riaz N. (2026). Nat. Cancer.

[6] Zhao H. (2021). Signal Transduct. Targeted Ther..

[7] Zhao L.Y. (2023). Signal Transduct. Targeted Ther..

[8] Gigi E. (2025). Nat. Cancer.

[9] Mountcastle S.E. (2020). J. Oral Microbiol..

[10] Sousa M.G.C. (2025). Front. Bioeng. Biotechnol..

[11] Buzhor M.G. (2026). Adv. Healthcare Mater..

[12] Gan Z. (2023). Bioact. Mater..

[13] Fernando K. (2021). Biomater. Sci..

[14] Bhusari S. (2022). Adv. Sci. (Weinh.).

[15] Vlachogiannis Georgios (2018). Science.

[16] Yuan C. (2022). Pharmacol. Ther..

[17] Vétizou M. (2015). Science.

[18] Rubinstein M.R. (2013). Cell Host Microbe.

[19] Park E.M. (2022). Nat. Med..

[20] Routy B. (2018). Science.

[21] Aghamajidi A., Maleki Vareki S. (2022). Cancers (Basel).

[22] Dominique G.M. (2024). Aging Cancer.

[23] Picardo S.L. (2019). Crit. Rev. Oncol. Hematol..

[24] Teng H. (2023). Cancer Cell.

[25] Singh V. (2018). Cell.

[26] Mohseni A.H. (2023). Cell Death Dis..

[27] Cao Y. (2024). Signal Transduct. Targeted Ther..

[28] Narunsky-Haziza L. (2022). Cell.

[29] Nejman D. (2020). Science.

[30] Ghaddar B. (2022). Cancer Cell.

[31] Yang L. (2023). Signal Transduct. Targeted Ther..

[32] Dominique G.M. (2024). Aging Cancer.

[33] Lehouritis P. (2015). Sci. Rep..

[34] Seano G. (2013). Blood.

[35] Palrasu M. (2022). PLoS Pathog..

[36] Moskal K. (2025). Int. J. Cancer.

[37] Gabelein C.G. (2022). ACS Synth. Biol..

[38] Nunes A.S. (2019). Biotechnol. Bioeng..

[39] Kular J.K. (2014). J. Tissue Eng..

[40] Nayak P. (2023). Cancers (Basel).

[41] Kasper S.H. (2020). Sci. Rep..

[42] Han S. (2020). Int. J. Mol. Sci..

[43] Sato T. (2009). Nature.

[44] Salahudeen A.A., Kuo C.J. (2015). Nat. Med..

[45] Aguilar C. (2021). Exp. Mol. Med..

[46] Sato T. (2011). Gastroenterology.

[47] Bartfeld S. (2015). Gastroenterology.

[48] Engevik M.A. (2015). Am. J. Physiol. Gastrointest. Liver Physiol..

[49] Leslie J.L. (2015). Infect. Immun..

[50] Pleguezuelos-Manzano C. (2020). Nature.

[51] Maschmeyer I. (2015). Lab Chip.

[52] Ronaldson-Bouchard K. (2022). Nat. Biomed. Eng..

[53] Kim S. (2016). Lab Chip.

[54] Yu J. (2022). Nano Converg..

[55] Park D. (2019). Front. Immunol..

[56] Lou J., Mooney D.J. (2022). Nat. Rev. Chem.

[57] Berry S.B. (2017). Lab Chip.

[58] Takahashi R. (2024). Adv. Mater. Technol..

[59] Ko J. (2019). Lab Chip.

[60] Shah P. (2016). Nat. Commun..

[61] Shin W. (2019). Front. Bioeng. Biotechnol..

[62] Kim H.J. (2012). Lab Chip.

[63] Shin Y.C. (2023). Nat. Rev. Bioeng..

[64] Lee J. (2024). Adv. Sci. (Weinh.).

[65] Jalili-Firoozinezhad S. (2019). Nat. Biomed. Eng..

[66] Subedi N. (2021). Sci. Rep..

[67] Henry O.Y.F. (2017). Lab Chip.

[68] Chen C. (2023). MedComm.

[69] Muthubharathi B.C. (2021). Mol. Omics.

[70] Freitas P. (2023). Sci. Rep..

[71] Hutmacher D.W. (2010). Nat. Mater..

[72] Saraswathibhatla A. (2023). Nat. Rev. Mol. Cell Biol..

[73] Liu X. (2024). Biosensors (Basel).

[74] Shabib M. (2026). ACS Appl. Bio Mater..

[75] Farrell L. (2025). Cells.

[76] Provenzano P.P. (2008). BMC Med..

[77] Lee K.Y., Mooney D.J. (2012). Prog. Polym. Sci..

[78] Clerkin S. (2025). Biomaterials.

[79] Bessot A. (2023). Adv. Healthcare Mater..

[80] Zhou D. (2025). Front. Bioeng. Biotechnol..

[81] Nikitovic D. (2013). Biomed Res. Int..

[82] Alsharabasy A.M., Pandit A. (2024). Tissue Eng. Part. C Method..

[83] Krutty J.D. (2016). Curr. Opin. Biotechnol..

[84] Song Y. (2025). Adv. Healthcare Mater..

[85] Pradhan S., Slater J.H. (2019). Biomaterials.

[86] Rafaeva M. (2022). Adv. Healthcare Mater..

[87] Butcher D.T. (2009). Nat. Rev. Cancer.

[88] Levental K.R. (2009). Cell.

[89] Jiang T. (2019). Biomaterials.

[90] Ermis M. (2023). Bioact. Mater..

[91] Peela N. (2016). Biomaterials.

[92] Yue X. (2018). Biomaterials.

[93] Montesano R. (1983). J. Cell Biol..

[94] Fischbacha C. (2009). Proc. Natl. Acad. Sci. USA.

[95] Cross V.L. (2010). Biomaterials.

[96] Verbridge S.S. (2010). Tissue Eng. Part A.

[97] Zhu C. (2026). Adv. Mater..

[98] Monteiro C.F. (2024). Int. Mater. Rev..

[99] Liu X. (2021). Microsyst. Nanoeng..

[100] Malik M. (2021). Front. Cell Dev. Biol..

[101] Lee T. (2026). Microsyst. Nanoeng..

[102] Udayasuryan B. (2022). Sci. Signal..

[103] Ronaldson-Bouchard K. (2022). Adv. Drug Deliv. Rev..

[104] Arora S. (2021). Front. Microbiol..

[105] Tuson H.H. (2012). Mol. Microbiol..

[106] Auer G.K. (2016). Cell Syst..

[107] Tan L.L. (2022). Carbohydr. Polym..

[108] Khodaei D. (2020). Food Biosci..

[109] Sun J.Y. (2012). Nature.

[110] Harimoto T. (2026). Science.

[111] Bettegowda C. (2003). Proc. Natl. Acad. Sci. USA.

[112] Hayashi M. (2023). Cancer Sci..

[113] Duong M.T. (2019). Exp. Mol. Med..

[114] Nguyen D.H. (2023). Nat. Commun..

[115] Din M.O. (2016). Nature.

[116] Chowdhury S. (2019). Nat. Med..

[117] Etter E.L. (2026). RSC Pharm.

[118] Yuan L. (2022). Commun. Biol..

[119] Discher D.E. (2005). Science.

[120] Sunyer R. (2016). Science.

[121] Wullkopf L. (2018). Mol. Biol. Cell.

[122] Wang L. (2023). bioRxiv.

[123] Rodesney C.A. (2017). Proc. Natl. Acad. Sci. U. S. A..

[124] Gordon V.D., Wang L. (2019). J. Cell Sci..

[125] Dupont S. (2011). Nature.

[126] Morad G. (2025). Nat. Med..

[127] Schorr L. (2023). npj Biofilms Microbiomes.

[128] Lin L., Zhang D. (2025). Front. Oncol..

[129] Huang J. (2023). Front. Cell. Infect. Microbiol..

[130] Liu X. (2021). Biomaterials.

[131] Aparicio-Yuste R. (2022). Front. Cell Dev. Biol..

[132] Wang L. (2023). npj Biofilms Microbiomes.

[133] Karim M.A. (2026). ACS Biomater. Sci. Eng..

[134] Zhang M., Zhang B. (2025). Exp. Hematol. Oncol..

[135] Sepich-Poore G.D. (2021). Science.

[136] Correa-Oliveira R. (2016). Clin. Transl. Immunol..

[137] Huang C. (2023). Comput. Struct. Biotechnol. J..

[138] Li S. (2023). Front. Nutr..

[139] Athari S.S. (2019). Signal Transduct. Targeted Ther..

[140] Wu Y.H. (2023). Theranostics.

[141] Liang C. (2026). Biomater. Res..

[142] Jang M.J. (2011). Vaccine.

[143] Liu L. (2022). J. Exp. Clin. Cancer Res..

[144] Maddineni S. (2022). J. Immunother. Cancer.

[145] Gopalakrishnan V. (2018). Science.

[146] Sivan Ayelet (2015). Science.

[147] Gurbatri C.R. (2022). Science.

[148] Zhu C. (2024). Signal Transduct. Targeted Ther..

[149] Mark B., Meads R.A. G.a.W.S. D. (2009). Nat. Rev. Cancer.

[150] Junttila M.R., de Sauvage F.J. (2013). Nature.

[151] Riquelme E. (2019). Cell.

[152] LaGory E.L., Giaccia A.J. (2016). Nat. Cell Biol..

[153] Ayuso J.M. (2022). Nat. Commun..

[154] Luke J.J. (2023). Clin. Cancer Res..

[155] Lynch J.P. (2022). Trends Pharmacol. Sci..

[156] Ma G. (2026). Nat. Commun..

[157] Chen W. (2022). Adv. Drug Deliv. Rev..

[158] Loeffler M. (2009). Cancer Immunol. Immunother..

[159] Zhang Y. (2023). Biomaterials.

[160] Le D.T. (2015). J. Clin. Oncol..

[161] Tsujikawa T. (2020). Clin. Cancer Res..

[162] Matthew E. Griffin (2021). Science.

[163] Zhang S. (2025). Mol. Biomed..

[164] Zhou X. (2022). Front. Oncol..

[165] Li M. (2024). Biomater. Res..

[166] Xiao M. (2024). View.

[167] Yang Q. (2024). J. Nanobiotechnol..

[168] de Haan L. (2021). Int. J. Mol. Sci..

[169] Ali Z. (2022). J. Exp. Clin. Cancer Res..

[170] Gao C. (2023). ACS Nano.

[171] Wang M. (2024). Nat. Biotechnol..

[172] Wang X. (2026). Biomaterials.

[173] Han Z.Y. (2024). Nat. Commun..

[174] Wong-Rolle A. (2022). J. Immunother. Cancer.

[175] Lotstedt B. (2024). Nat. Biotechnol..

[176] Saarenpaa S. (2024). Nat. Biotechnol..

[177] Sun C. (2023). Nat. Commun..

[178] Wu H. (2025). iScience.

[179] Lavrador P. (2021). EBioMedicine.

[180] Pisani M. (2025). Nat. Commun..

